# Influence of Urban Landscapes on Population Dynamics in a Short-Distance Migrant Mosquito: Evidence for the Dengue Vector *Aedes aegypti*

**DOI:** 10.1371/journal.pntd.0000634

**Published:** 2010-03-16

**Authors:** Ryan R. Hemme, Clayton L. Thomas, Dave D. Chadee, David W. Severson

**Affiliations:** 1 Department of Biological Sciences, Eck Institute for Global Health, University of Notre Dame, Notre Dame, Indiana, United States of America; 2 Department of Life Sciences, University of the West Indies, St. Augustine, Trinidad and Tobago, West Indies; Duke University-National University of Singapore, Singapore

## Abstract

**Background:**

Dengue viruses are endemic across most tropical and subtropical regions. Because no proven vaccines are available, dengue prevention is primarily accomplished through controlling the mosquito vector *Aedes aegypti*. While dispersal distance is generally believed to be ∼100 m, patterns of dispersion may vary in urban areas due to landscape features acting as barriers or corridors to dispersal. Anthropogenic features ultimately affect the flow of genes affecting vector competence and insecticide resistance. Therefore, a thorough understanding of what parameters impact dispersal is essential for efficient implementation of any mosquito population suppression program. Population replacement and genetic control strategies currently under consideration are also dependent upon a thorough understanding of mosquito dispersal in urban settings.

**Methodology and Principal Findings:**

We examined the effect of a major highway on dispersal patterns over a 2 year period. *A. aegypti* larvae were collected on the east and west sides of Uriah Butler Highway (UBH) to examine any effect UBH may have on the observed population structure in the Charlieville neighborhood in Trinidad, West Indies. A panel of nine microsatellites, two SNPs and a 710 bp sequence of mtDNA cytochrome oxidase subunit 1 (*CO1*) were used for the molecular analyses of the samples. Three *CO1* haplotypes were identified, one of which was only found on the east side of the road in 2006 and 2007. AMOVA using mtCO1 and nuclear markers revealed significant differentiation between the east- and west-side collections.

**Conclusion and Significance:**

Our results indicate that anthropogenic barriers to *A. aegypti* dispersal exist in urban environments and should be considered when implementing control programs during dengue outbreaks and population suppression or replacement programs.

## Author Summary

Worldwide, 2.5 billion people are at risk for dengue infection, with no vaccine or treatment available. Thus dengue prevention is largely focused on controlling its mosquito vector, *Aedes aegypti*. Traditional mosquito control approaches typically include insecticide applications and breeding site source reduction. Presently, novel dengue control measures including the sterile insect technique and population replacement with dengue-incompetent transgenic mosquitoes are also being considered. Success of all population control programs is in part dependent upon understanding mosquito population ecology, including how anthropogenic effects on the urban landscape influence dispersal and expansion. We conducted a two year population genetic study examining how a major metropolitan highway impacts mosquito dispersal in Trinidad, West Indies. As evidenced by significant differentiation using both nuclear and mitochondrial DNA sequences, the highway acted as a significant barrier to dispersal. Our results suggest that anthropogenic landscape features can be used effectively to enhance population suppression/replacement measures by defining mosquito control zones along recognized landscape barriers that limit population dispersal.

## Introduction

Anthropogenic assisted invasions by non-indigenous insect vectors of human disease have and will continue to have profound effects on global health [1]. In addition, anthropogenic land use changes can represent primary drivers of infectious disease epidemics and significantly alter disease transmission dynamics [2]. The dengue and yellow fever vector mosquito, *Aedes aegypti*, is a remarkably successful invasive species. A highly anthropophilic form likely emerged in North Africa within the past 2–4 thousand years and has subsequently been transported via human efforts to most subtropical and tropical regions worldwide [3],[4].

Approximately two-fifths of the world's population is at risk for dengue infection and an estimated 500,000 people are affected by dengue hemorrhagic fever (DHF) annually, with fatality rates exceeding 20% when proper treatment is unavailable [5]. Dengue is widely distributed in the tropics, occurring in Central and South America, South and Southeast Asia, Africa, the Caribbean, and Pacific regions [6]. During the past 45 years the incidence of dengue infection has steadily increased throughout the globe as greater numbers of people permanently migrate to cities with continued growth and urbanization [7].

*Aedes aegypti* population dynamics in urban areas is subject to daily as well as seasonal meteorological variability [8]. The interaction between temperature, relative humidity and rainfall impact adult survival and availability of oviposition sites. The goal of *A. aegypti* control programs is to reduce the population density of adult mosquitoes below a critical threshold where epidemic dengue transmission is unlikely to occur [9]. Vector population suppression programs most often involve the elimination or insecticide treatment of larval habitats that are typically man-made containers located within or around houses. During epidemic outbreaks, ultra low volume (ULV) spraying of insecticides is often used as an emergency control measure to reduce the adult mosquito population [10]. Critical to the long-term success of any *A. aegypti* population suppression method is the influence of dispersion patterns of adult mosquitoes. A greater understanding of factors limiting adult dispersal would allow health agencies to be more efficient in allocating resources to vector control programs. Moreover, considerable interest exists in developing novel dengue control strategies through the development of genetically modified *A. aegypti* incapable of transmitting dengue virus (DENV) and their subsequent introduction into the field as part of a population replacement program [11],[12]. A thorough understanding of dispersal behavior in urban environments is essential to successful implementation of any control strategy.

Population structure of *A. aegypti* is complex, varies by region and scale, and can be influenced by environment and geography [13]–[20]. Urban estimates of genetic differentiation have varied in part due to environmental conditions and dispersal patterns [21]–[24]. Typically, adult *A. aegypti* mosquitoes travel relatively short distances of up to ∼100 m, although longer dispersal estimates of ∼800 m have been observed [25]–[29]. In Queensland, Australia, a mark-release-recapture study reported that *A. aegypti* would readily cross smaller, quieter roads, but significantly fewer crossed a major highway near the release point, and concluded that busy roads may have impeded dispersal [30]. Similar observations were made with bumblebees (*Bombus impatiens* and *B. affinis*), where Bhattacharya *et al.* [31] reported high fidelity between the bumblebees and their foraging sites and that they would rarely cross nearby roads or railways. These observations indicate the possibility that habitat fragmentation due to roads or other anthropogenic environmental manipulations may act as significant barriers to migration of *A. aegypti* and other insects.

In urban environments anthropogenic landscape features can result in habitat fragmentation and thereby influence dispersal patterns of mosquitoes. Ultimately, these features can affect the flow of genes conditioning vector competence and insecticide resistance. In the current study, we report evidence of limited *A. aegypti* movement across an expansive 4 lane split highway in an urban environment in Trinidad, West Indies, as evidenced by mitochondrial and nuclear molecular analyses.

## Materials and Methods

### Sample collections

**Figure 1:**
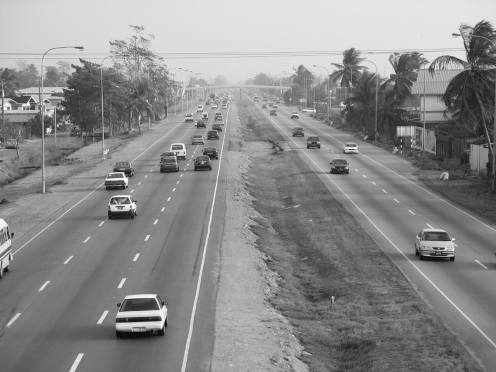
Uriah Butler Highway. Samples were collected along an ∼900 m stretch of the highway. The distance across the highway is ∼65 m.

**Figure 2:**
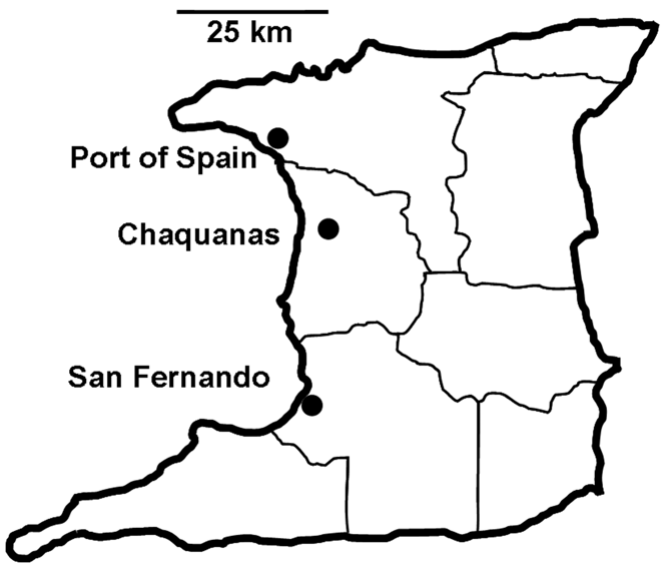
Collection sites were located along Uriah Butler Highway in the Charlieville neighborhood of Chagaunas, Trinidad, West Indies. This is the major north-south highway from Port of Spain to San Fernando.

*A. aegypti* larvae were collected within urban breeding sites along a 900 m length of Uriah Butler Highway (UBH) on the east and west sides in the Charlieville neighborhood of Chauguanas. UBH is the major north-south highway in western Trinidad, extending from San Fernando in the south to east of Port of Spain (Figs. 1 & 2). The distance between buildings on the east and west sides of UBH ranged from ∼80 m to ∼130 m and the two sides were connected by a walking overpass and on-off ramps on both ends of the sampling area. Charlieville is a diverse urban neighborhood with mixed commercial, industrial, and residential buildings clustered closely together.

Larvae were collected during October of 2006 and 2007 with assistance from field technicians within the Insect Vector Control Division at the Ministry of Health. In 2006, larvae were collected from 18 larval habitat sites and in 2007 collections were taken from nine sites. Our samples were collected from diverse, but typical, larval habitats including water storage drums, folded sheets of commercial plastic, small buckets, and neglected and disused auto parts. Larvae were preserved in ethanol, and carried to the University of Notre Dame for genotyping.

### DNA extraction

DNA was extracted from the mosquito samples using a standard phenol-chloroform method [32]. In 2006, 147 larvae were genotyped; 58 from the western side of UBH and 89 on the eastern side while in 2007, DNA was extracted from a total of 83 larvae; 33 from the west and 50 from the east.

### Mitochondrial gene amplification and haplotype determination

A 710-basepair region of the cytochrome c oxidase subunit I gene (*CO1*) was amplified using *CO1*-specific universal primers [33]. Five µl of template DNA was amplified by polymerase chain reaction (PCR) in a 25.0 µl reaction containing 1X Taq buffer (10 mM KCl, 2 mM Tris, pH 9.0, 0.02% TritonX), 1.5 mM MgCl_2_, 0.4 mM each dATP, dCTP, dGTP, dTTP, 5 pmoles of each primer, and 1 U Taq DNA polymerase. The thermocycle conditions were 94°C for 1 min, 4 cycles at 94°C for 1 min, 45°C for 1.5 min, followed by 34 cycles at 94°C for 1 min, 50°C for 1.5 min, and 72°C for 1 min with a final extension period at 72°C for 5 minutes.

Haplotypes were identified by examining banding patterns using Single Strand Conformation Polymorphism (SSCP) as per [34]. Briefly, 5.0 µl of PCR product was mixed with 3.0 µl of Denaturing Loading Mix (DLM), that consisted of 0.1 ml of 1N NaOH, 9.5 ml formamide, 0.005 g of bromophenol blue, 0.005 g of xylene cyanol, and brought up to 10 ml with ddH_2_0. The mixture was denatured at 95°C for 5 min and snap cooled on ice. Approximately 7.0 µl of PCR product-DLM were used for electrophoresis on 42×33 cm 5% polyacrylamide gels for 3–4 hours at 30 milliamps. Gels were stained using silver nitrate solution with protocol adapted from Promega's *GenePrint*® STR Systems (Promega U.S., Madison, WI) to visualize DNA banding patterns. Confirmation of haplotypes was accomplished by sequencing 5–10 individuals of each haplotype from both strands using the same *CO1* primers.

### Nuclear marker amplification and polymorphism detection

A total of 11 markers including nine microsatellite loci [35],[36] and two Single Nucleotide Polymorphism (SNP) loci [37] were used for genotyping. PCR amplification was performed with genomic DNA isolated from individual mosquitoes in 25 µl volumes as described above. PCR reactions for microsatellite loci AC2, AG2, AG7, A10, B19, CT2, H08, M201, M313 were performed under the following conditions: 94°C for five minutes, followed by 30 cycles of 94°C for 1 min, 60°C anneal for 1 min, 72°C extension for 2 min, and a final 72°C extension for 10 min. PCR conditions for SNP loci LF178 and RT6 were performed at: 94°C for 5 minutes, followed by 39 cycles at 94°C for 20 sec, 55°C for 20 sec, and 72°C for 30 sec, and a final 72°C extension for 10 min. SNP products LF178 and RT6 were digested with *Rsa*I and *Mnl*I respectively and size fractionated in 3% agarose gels and visualized with ethidium bromide under UV light.

Polymorphisms in microsatellite loci were resolved and analyzed using a Beckman-Coulter CEQ8000 and Beckman-Coulter CEQ8000 software. Briefly, microsatellites were amplified using dye-labeled primers (Sigma Proligo, Sigma-Aldrich Inc., St. Louis, MO) and pooled into groups of 3 loci. Pools consisted of 0.4 µl of 400 bp standard, and 30.0 µl of standard loading solution (Beckman-Coulter Inc., Fullerton, CA) with 1.0 µl of diluted amplified product added to each well.

### Data analysis

Conformance with Hardy-Weinberg equilibrium (HWE), gametic disequilibrium between pairs of loci in each population, and inbreeding coefficients (F_IS_) were computed on FSTAT version 2.9.3.2 [38]. Presence of null alleles was examined using Micro-Checker [39]. Mitochondrial sequences were aligned using SEQMAN from the Lasergene package (DNASTAR Inc., Madison, WI) and analyzed using DnaSP [40]. Population structure was examined using locus by locus Analysis of Molecular Variance (AMOVA) for nuclear markers and mtDNA haplotypes were analyzed using standard AMOVA on Arlequin version 3.1 [41].

## Results

### mtDNA CO1 polymorphism

**Figure 3:**
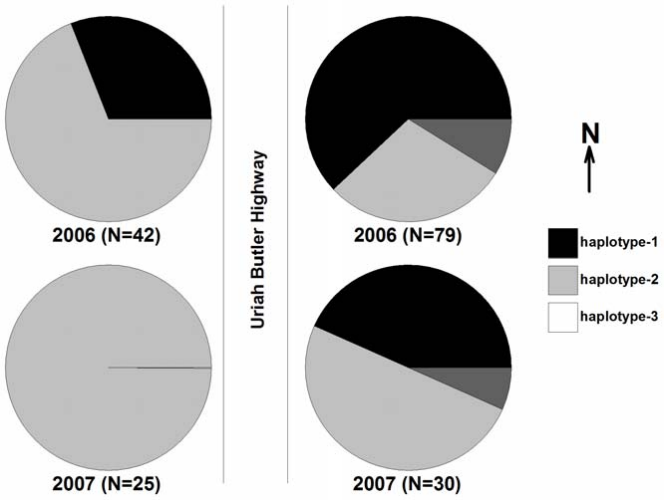
Frequency of unique *COI* mtDNA haplotypes recovered from the Charlieville neighborhood on the east and west side of Uriah Butler Highway in 2006 and 2007.

The mitochondrial *CO1* gene showed sequence polymorphisms among the samples. A total of 3 haplotypes were identified within the 177 individuals in our test samples (Fig. 3). Mitochondrial *CO1* haplotypes-1 and 2 were the most common across populations and years, accounting for ∼42% and ∼52% of total known haplotypes. Haplotype-3 comprised ∼5% of the total, but was unique to the eastern side of the road in 2006 and 2007 samples and was never detected on the western side of UBH. Haplotype-2 was the only haplotype detected from the 2007-west population (Fig. 3). Haplotype frequencies were compared spatially (east compared to west) and temporally (2006 compared to 2007). There was significant differentiation between mosquitoes collected on the east side of the road and those collected on the west side of the road in 2006 and 2007 (Table 1). Temporally, the 2006-west population differed from the 2007-west population, however on the east side of the road there was no genetic differentiation between years. Estimated F_ST_ values were moderate to large, ranging from 0.042 to 0.390. Our data suggested relatively lower F_ST_ values for spatial samples than temporal samples. The east and west side samples show F_ST_ 0.172 (year 2006) and 0.390 (year 2007) and the 2006 and 2007 samples show F_ST_ 0.249 (east) and 0.49 (west).

**Table 1:** Analysis of molecular variance using mtDNA CO1 haplotypes.

Source	d.f.	Sum of squares	Variance components	Variation (%)	F_ST_
Between east & west, **2006**	1	39.8	0.6644	17.2	0.172†
Within directions	120	384.5	3.2044	82.8	
Total	121	424.3	3.8688		
Between east & west, **2007**	1	36.1	1.2510	39.0	0.390*
Within directions	53	103.6	1.9554	61.0	
Total	54	139.7	3.2064		
Between 2006 & 2007, **east**	1	10.1	0.1516	4.22	0.042♦
Within years	108	371.5	3.4395	95.78	
Total	109	381.6	3.5911		
Between 2006 & 2007, **west**	1	19.5	0.5655	23.9	0.240‡
Within years	65	116.7	1. 7952	76.1	
Total	66	136.2	2.3607		

Probability (random value ≥ observed value) (10,100 permutations).

†0.00040+/−0.00019.

*0.00000+/−0.00000.

♦0.06891+/−0.00250.

‡0.00238+/−0.00051.

### Nuclear marker polymorphism

Five out of 44 tests (11%) were significant for deviation from Hardy-Weinberg equilibrium after Bonferroni correction (Table 2). Deviations in expected heterozygosity were due to heterozygosity deficits in locus B19 (2006-east and 2006-west collections), and locus M313 (2006-east and 2007-east collections). Deviation at locus M201 in the 2007-west population was a result of heterozygote excess. Null alleles were identified at loci LF178 and RT6 in the 2006-east and 2007-east populations, respectively and in locus A10 in both 2007 populations, but the loci were in HW equilibrium. Evidence for null alleles was present in locus M313 in all but the 2007-west population, and locus B19 had high levels of null alleles in all populations, and both B19 and M313 deviated from HW equilibrium. Gametic disequilibrium analysis revealed significant disequilibrium between loci H08 and A10 and loci H08 and AG7. The H08 and AG7 loci are physically linked on chromosome II, while locus A10 is located on chromosome III. Due to the presence of null alleles at loci B19, M313, and A10, and gametic disequilibrium with locus H08, these markers were removed prior to AMOVA.

**Table 2:** Summary of variation at 9 microsatellite and 2 SNP loci by collection.

Chromosome	Locus		Population			
			2006-East	2006-West	2007-East	2007-West
1	AC2LF178*RT6*	NF_IS_NF_IS_NF_IS_	87−0.17076−0.154820.420	50−0.031390.177440.333	280.285190.74146−0.071	270.47512−0.100330.283
2	AG2AG7CT2H08	NF_IS_NF_IS_NF_IS_NF_IS_	830.031700.077840.10381−0.031	460.007360.17847−0.13344−0.059	460.054360.083360.041380.028	33−0.08127−0.26526−0.009320.203
3	A10B19M201M313	NF_IS_NF_IS_NF_IS_NF_IS_	820.11570**0.294**75−0.21148**0.801**	450.13140**0.445**45−0.203280.441	390.382430.26937−0.28638**0.537**	240.462270.36433−**0.641**330.305

N, number of individuals; F_IS_, inbreeding coefficient.

Bold denotes significant departure from HW after Bonferroni correction.

*denotes SNPs.

**Figure 4:**
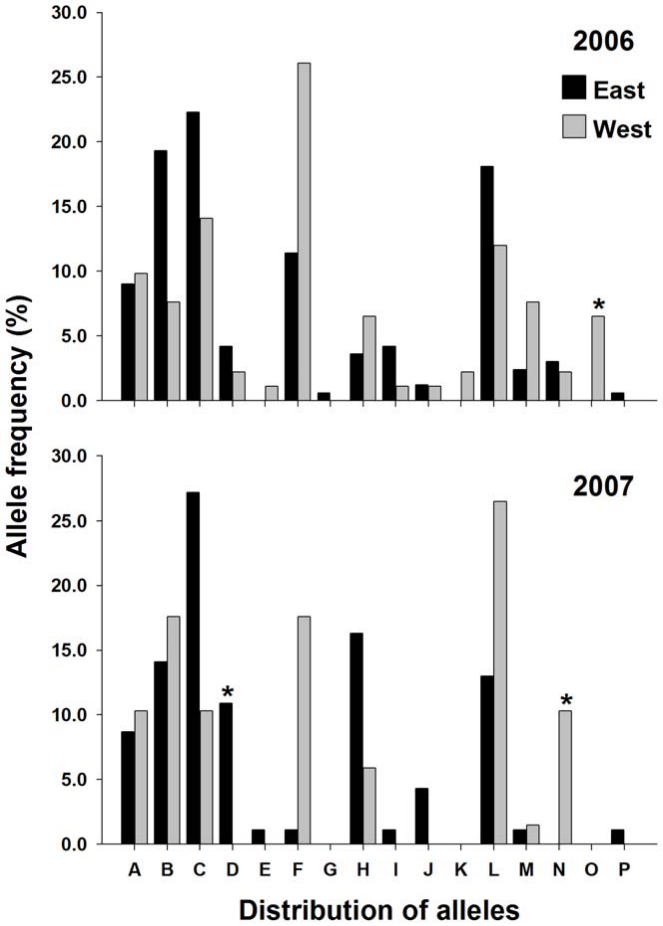
Distribution of alleles for microsatellite locus AG2 in 2006 and 2007. *Denotes private alleles with a frequency of >5%.

**Figure 5:**
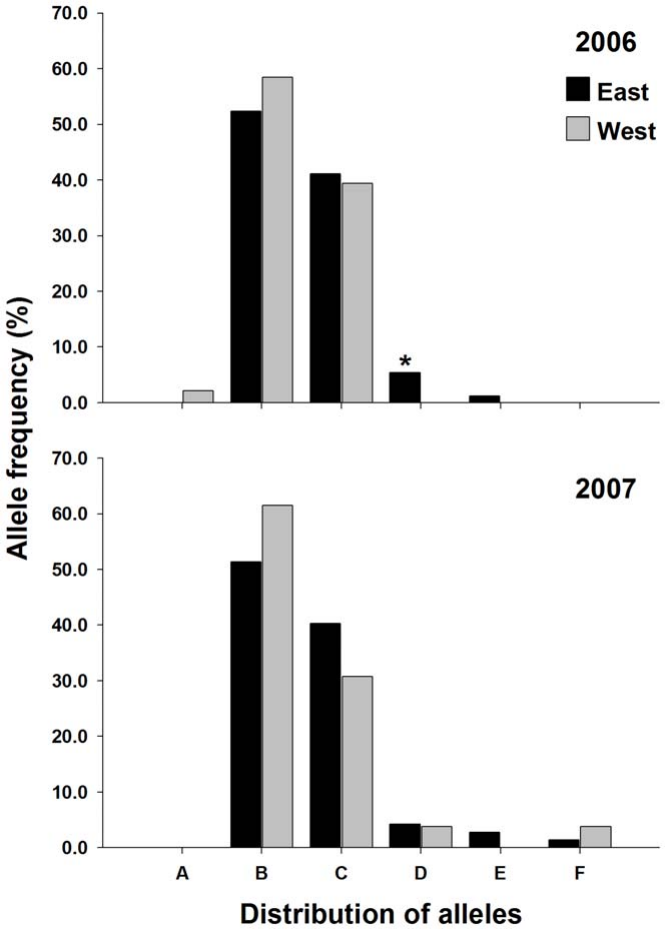
Distribution of alleles for microsatellite locus CT2 in in 2006 and 2007. *Denotes private alleles with a frequency of >5%.

**Figure 6:**
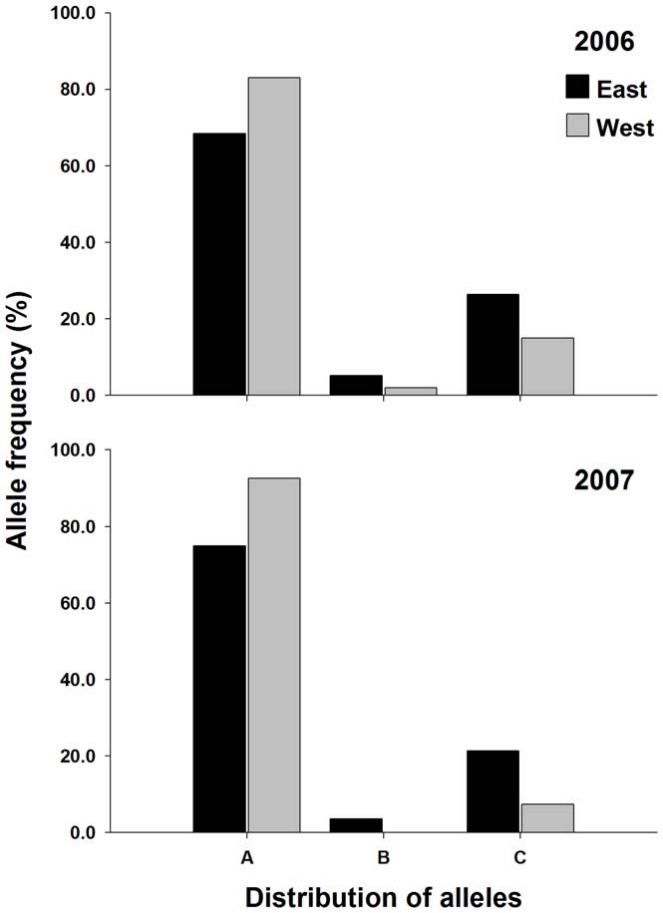
Distribution of alleles for microsatellite locus AC2 in 2006 and 2007.

Four of the five microsatellites had private alleles that were specific to either the 2006-east, 2006-west, 2007-east or 2007-west collections. Neither SNP locus had private alleles. Markers AG2 and CT2 had alleles that were present in a population at a frequency between 5–10% (Fig. 4 & 5). In marker AG2, 9 of the 16 alleles were private to at least one collection, however most had a frequency <3%. Allele AG2-O was present only in the 2006-west collection at 6.5%. Allele AG2-D was present in 2006-east, 2006-west, and in 2007 was only present in the 2007-east collection at a frequency of 10.9% (Fig. 4). Similarly allele AG2-N was present in both 2006 collections (east and west), but unlike allele AG2-D was absent in the 2007-east collection and present in the 2007-west collection at 10.3% (Fig. 4). Six alleles were found in CT2, 3 were private to at least one collection (Fig. 5). Allele CT2-D was present in the 2006-east (5.4%) collection, 2007-east (4.2%), and 2007-west (3.9%), but was not present in the 2006-west collection (Fig. 4). In the remaining 2 microsatellites (AC2 and AG7) alleles that were private in at least one collection existed, but the frequency ranged from ∼2% to ∼4% (Fig. 5 & 6). Overall, the amount of variation contained between populations was small, ranging from 0.2% to 1.4% of the total variation. AMOVA found small but significant F_ST_ estimates between collections on the east and west side of UBH in 2006 and 2007, ranging from 0.011 to 0.021 (Table 3).

**Table 3:** Analysis of molecular variance using nuclear microsatellite and SNP markers.

Source	Sum of squares	Variance components	Variation (%)	F_ST_
Between east v. west, **2006**	3.8	0.0175	1.1	0.011*
Within directions	406.2	1.6558	98.9	
Total	410.0	1.6733		
Between east v. west, **2007**	10.4	0.0198	1.2	0.021***
Within directions	614.9	1.6407	98.8	
Total	625.3	1.6605		
Between 2006 v. 2007, **east**	1.9	0.0033	0.2	0.002
Within years	390.5	1.6934	99.8	
Total	392.5	1.6967		
Between 2006 v. 2007, **west**	3.3	0.0222	1.4	0.014*
Within years	224.4	1. 5579	98.6	
Total	227.7	1.5801		

*P<0.05,

**P<0.01,

***P<0.001.

## Discussion

*Aedes aegypti* is highly adapted to a peridomestic environmental niche which has enabled it to spread throughout most large tropical cities in the world [42]. After confirmation that *A. aegypti* was the primary vector of yellow fever virus and dengue virus, disease prevention programs focused on the control of the mosquito vectors [43]. The pinnacle of dengue control began with the decision by PAHO to eradicate *A. aegypti* from the western hemisphere using a top down control structure and effective use of insecticides [42],[44],[45]. Incidences of *A. aegypti* transmitted diseases were greatly reduced along with the distribution of *A. aegypti* in the late 1950s to mid-1970s. However, in the late-1970s control programs were disbanded in part to financial considerations and the realization that unless a global campaign to eradicate *A. aegypti* was undertaken any attempts to eliminate the mosquito from the Americas would be unsuccessful due to the increased frequency and speed of air travel and other transportation options capable of transporting *A. aegypti* eggs and adults [46],[47]. Further complicating eradication efforts was the emergence of resistance to insecticides in the mid-1950s [46]. As a result in areas where *A. aegypti* was once eliminated, reinfestation and outbreaks of dengue eventually followed where vigilant vector surveillance and control was not implemented [43],[47],[48]. In addition, the availability of a highly effective vaccine for yellow fever likely contributed to a decline in active mosquito surveillance programs in areas certified as *A. aegypti* free.

Our micro-geographic analysis of genetic variability in *A. aegypti* from Trinidad was studied using microsatellite and SNP nuclear markers, and mitochondrial *CO1* sequences. The distribution of the 3 *CO1* haplotypes is strongly indicative of UBH acting as a barrier to dispersal. We were unable to detect haplotype-3 on the west side of the road in either 2006 or 2007. This is interesting because the distance between collections on the east and west side of UBH ranged from ∼80 m to ∼130 m, which given dispersal estimates ranging from 100 m to 800 m, should not have limited adult dispersal potential and mosquitoes with haplotype-3 would likely be expected to have colonized the west side of UBH unless they were unable to successfully transect the highway. F_ST_ estimates from AMOVA also revealed significant differentiation between populations collected on the east and west side of UBH in 2006 and 2007. Results from nuclear marker analysis therefore showed the same pattern of differentiation as the mitochondrial sequence data; however the magnitude of the F_ST_ and amount of variation was much lower (Table 3). Two explanations, neither mutually exclusive may explain the discrepancy in magnitude between the class of markers. The first is due to the preservation of diversity as a result of *A. aegypti* utilizing heterogeneous larval habitats in Trinidad. A large number of alternative habitats that are suitable for mosquito production may have gone undetected by surveyors in the Charlieville neighborhood as indicated by the large numbers of alleles detected (Fig. 4, 5, 6 & 7). Of the 25 containers from which we collected larvae, 10 were from containers other than water storage drums. In Trinidad water storage drums are the main source of *A. aegypti* production, however surveys have shown that *A. aegypti* mosquitoes will utilize a wide range of permanent and semi-permanent containers and the types of containers used can depend upon the degree of urbanization [49],[50]. The second explanation is the existence of homoplasy in the microsatellites which has been proposed as an explanation for observed differences in differentiation between SNP and mtDNA marker in *A. aegypti* populations in Venezuela [13]. If present, homoplasy would underestimate the amount of differentiation between the collections [51].

**Figure 7:**
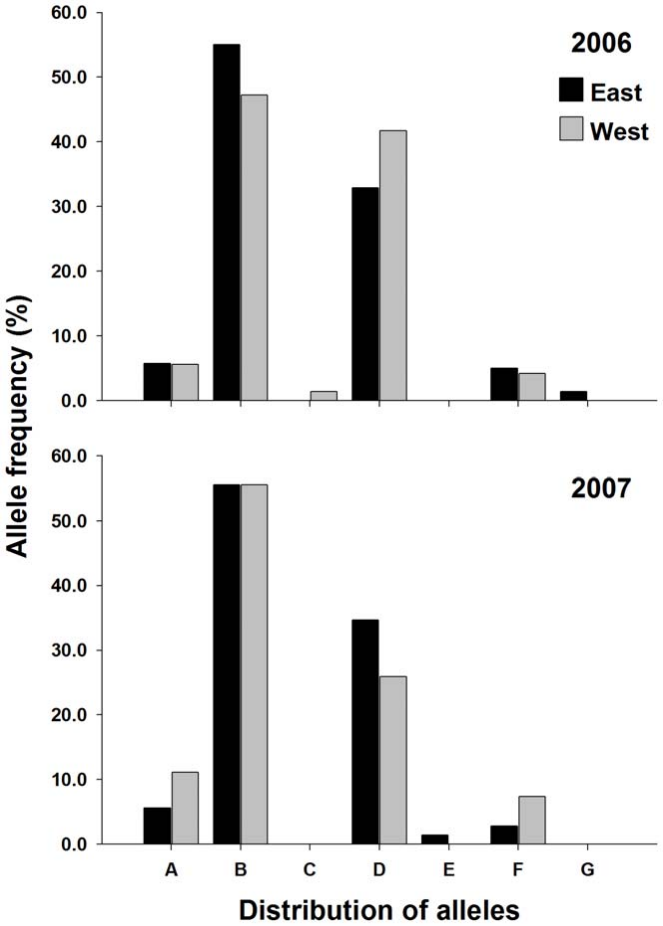
Distribution of alleles for microsatellite locus AG7 in 2006 and 2007.

We also observed temporal differences in haplotype frequency between 2006 and 2007, although no significant population structure was found between 2006 and 2007 on the east side of UBH (Table 1). In all four groups of mosquitoes there was a decrease in the frequency of *CO1* haplotype-1 and increase in haplotype-2 (Fig. 3). One possible explanation for this observation is that the resulting change in haplotype frequencies is a consequence of vector control efforts conducted periodically by the Ministry of Health in Trinidad. Changes in haplotype frequency could also be due to normal temporal fluctuations in the mosquito population. If the former is the primary cause affecting haplotype frequency one would expect to see reduced heterozygosity in the nuclear markers, which was not observed. Although there was not a physical barrier separating the 2006 and 2007 collections the significant differentiation observed between the 2006 west and 2007 west population was not unexpected, as previous examinations of temporal variation in *A. aegypti* populations have also reported changes in genetic differentiation over seasons, which were most likely due to changes in mosquito density, availability of oviposition sites, and the type of environment [21],[23],[52]. In Phnom Penh, Cambodia, genetic differentiation in *A. aegypti* populations was influenced by seasonality and environment type (urban vs. suburban vs. rural), with significant differentiation occurring within the city [23]. The authors suggested that ideal urban conditions, including an abundant supply of hosts and oviposition sites, limited the need for dispersal by adult mosquitoes. In suburban areas differentiation was in part dependent upon the physical environment with variations in human density, availability of running water, and rural versus residential developments impacting the population structure.

Dispersal range is an important aspect of dengue transmission and much research has been conducted attempting to determine how far *A. aegypti* adults travel, however large variations in daily and lifetime dispersal rates have been reported. Larger estimates of dispersal have reported mosquitoes traveling >800 m [29],[53]. Many studies using mark-release-recapture methods have reported a shorter flight range of *A. aegypti* [25], [54]–[56]. Examining mean distance traveled (MDT) and the flight range within which 50% (FR50) and 90% (FR90) of mosquitoes travel, as opposed to maximum distance traveled may be a more epidemiologically important parameter [29]. In a Kenyan village, McDonald and others [25] recaptured a majority of mosquitoes within the house they were released over 12 days. Marked mosquitoes released in a tire dump in New Delhi, India dispersal ranged between 50–200 m, but most were recaptured within 50 m of the release point [57]. Similarly, Muir and Kay [54] reported females having a MDT of 56 m and FR90 of 108 m. Released mosquitoes tended to cluster around houses with some dispersal towards adjacent houses and mosquitoes released on the perimeter of villages moved towards the center of the village [26], [29], [58]–[60]. The relatively large numbers and duration of DENV infected females captured in houses with confirmed dengue cases in Merida, Mexico may further indicate high fidelity between *A. aegypti* mosquitoes and place of pupal emergence [61].

Results from both classes of makers show strong evidence of limited gene flow across UBH, effectively fragmenting the populations on the east and west side of the highway. Mosquito dispersal patterns are nonrandom and influenced by environmental factors as reported by Sheppard et al. [62] and Hausermann et al. [63] in *A. aegypti* mosquitoes using mark-release-recapture methods. Furthermore, Chadee [64] indicated that prevailing weather patterns may potentially influence dispersion. Range of dispersal is dependent upon a mosquito's ability to remain in flight and the availability and abundance of shelter, food sources, hosts for blood meals and suitable oviposition sites [62]. Suitable host availability may reduce dispersal as reported by Suwonkerd et al. [65] where fewer *A. aegypti* mosquitoes exited a hut when a human host was present than with controls consisting of a dog or no host. Edman et al. [66] reported that when an abundance of suitable oviposition sites were available dispersion of female *A. aegypti* mosquitoes was reduced. Although the distance across the highway is well within dispersal estimates for *A. aegypti*, lack of cover and shade may have made UBH a harsh environment for mosquitoes to transect. This is supported by Tun-Lin et al. [67] who reported shade as a significant factor impacting the presence of *A. aegypti* in premise surveys and Russell et al. [30] reported that released *A. aegypti* dispersal patterns were nonrandom with more mosquitoes being recaptured along a corridor with heavy shading from trees and vegetation. Furthermore, oviposition sites were most likely minimal, even along peripheral ditches and nonexistent blood meal hosts may have dissuaded migration across UBH and prevented a stepping stone model of colonization from occurring over UBH.

Ecological features including accessible water and availability of oviposition sites, vegetation patterns, humidity, and housing density contribute to determining the distribution of *A. aegypti* mosquitoes. The effects of topographic features of urban environments are not fully understood, however Reiter et al. [27] noted that buildings were not an impediment to *A. aegypti* flight. Our results indicate that urban landscape features do contain barriers to dispersal, and thereby affect the population structure of mosquitoes. This information could be used by vector control agencies to more efficiently target mosquito populations for suppression. Control programs can divide an urban area into zones of control along landscape features that are large enough to impede dispersal. This technique allows for the possibility of local elimination of *A. aegypti* moquitoes, barring or at least minimizing re-infestation due to the active transportation of the mosquito. Furthermore, during dengue outbreaks control agencies can more accurately target areas of higher risk along these same control zones.

Difficulties in vaccine development [68] and the sequencing of the entire *A. aegypti* genome [69] have shifted some research efforts to preventing illness by developing applications that make use of transgenic mosquitoes incapable of transmitting the virus. Central to the successful use of transgenic mosquitoes to replace competent vector populations or to effect population suppression/elimination is a thorough understanding of *A. aegypti* bionomics, answering the basic questions of how many mosquitoes need to be released, where is the best place for them to be released, and when should they be released [70]. Results from early efforts using the sterile insect technique (SIT) to eliminate mosquito populations could have been improved or were negatively influenced by incomplete knowledge of adult mosquito dispersal behavior in *A. aegypti* and *Culex fatigans* [71],[72]. Anthropogenic landscape features may therefore have profound effects on the implementation of traditional as well as proposed novel genetic mosquito control programs. Yakob et al. [73] explored the dynamics of population suppression dynamics with SIT and insects engineered to carry a dominant lethal gene (RIDL). Mathematical models indicated that dispersion parameters for *A. aegypti* were fundamental in the success of replacement efforts and that enhanced connectivity treatment and peripheral populations could result in increased densities of wild-type mosquitoes as a consequence of SIT programs. Natural and anthropogenic barriers may actively influence, either positively or negatively depending on the strategy, the success of population replacement or population reduction by limiting the effective range of gene flow. Understanding the role of landscape features on population dispersal is likely critical to achieving success with any *A. aegypti* control strategy.
